# A pitfall in the reconstruction of fibre ODFs using spherical deconvolution of diffusion MRI data

**DOI:** 10.1016/j.neuroimage.2012.10.022

**Published:** 2013-01-15

**Authors:** G.D. Parker, D. Marshall, P.L. Rosin, N. Drage, S. Richmond, D.K. Jones

**Affiliations:** a Cardiff University Brain Research Imaging Centre (CUBRIC), School of Psychology, Cardiff University, UK; b School of Computer Science & Informatics, Cardiff University, UK; c School of Dentistry, Cardiff University, UK; d Neuroscience and Mental Health Research Institute, Cardiff University, UK

**Keywords:** Spherical harmonic deconvolution, Richardson–Lucy, MRI, Calibration, Tractography, Diffusion tensor imaging

## Abstract

Diffusion weighted (DW) MRI facilitates non-invasive quantification of tissue microstructure and, in combination with appropriate signal processing, three-dimensional estimates of fibrous orientation. In recent years, attention has shifted from the diffusion tensor model, which assumes a unimodal Gaussian diffusion displacement profile to recover fibre orientation (with various well-documented limitations), towards more complex high angular resolution diffusion imaging (HARDI) analysis techniques.

Spherical deconvolution (SD) approaches assume that the fibre orientation density function (fODF) within a voxel can be obtained by deconvolving a ‘common’ single fibre response function from the observed set of DW signals. In practice, this common response function is not known *a priori* and thus an estimated fibre response must be used. Here the establishment of this single-fibre response function is referred to as ‘calibration’. This work examines the vulnerability of two different SD approaches to inappropriate response function calibration: (1) constrained spherical harmonic deconvolution (CSHD)—a technique that exploits spherical harmonic basis sets and (2) damped Richardson–Lucy (dRL) deconvolution—a technique based on the standard Richardson–Lucy deconvolution.

Through simulations, the impact of a discrepancy between the calibrated diffusion profiles and the observed (‘Target’) DW-signals in both single and crossing-fibre configurations was investigated. The results show that CSHD produces spurious fODF peaks (consistent with well known ringing artefacts) as the discrepancy between calibration and target response increases, while dRL demonstrates a lower over-all sensitivity to miscalibration (with a calibration response function for a highly anisotropic fibre being optimal). However, dRL demonstrates a reduced ability to resolve low anisotropy crossing-fibres compared to CSHD. It is concluded that the range and spatial-distribution of expected single-fibre anisotropies within an image must be carefully considered to ensure selection of the appropriate algorithm, parameters and calibration. Failure to choose the calibration response function carefully may severely impact the quality of any resultant tractography.

## Highlights

► We compare two spherical deconvolution based fODF retrieval techniques. ► We examine fODF error under inappropriate response function calibration. ► CSHD produces errors as response and target diffusion profiles diverge. ► dRL is poor against low FA targets but adequate across white matter. ► Manuscript updated to cover orientational dependence of CSHD errors.

## Introduction

In recent years diffusion weighted MRI (DW-MRI) (Le Bihan et al., 1986) has become a valuable tool for clinical and experimental neuroscience research being the only methodology for characterising tissue microstructure *in vivo*. To date, diffusion tensor MRI (DT-MRI) (Basser et al., 1994) is the most widely applied technique, providing useful quantitative microstructural indices such as mean diffusivity (MD) and fractional anisotropy (FA) (Pierpaoli and Basser, 1996a, 1996b), and basic tract reconstruction schemes based on the orientation of the principal eigenvector (Basser et al., 2000). However, the utility of DT-MRI is limited since the assumption of an ellipsoidal diffusion profile (the tensor model) prohibits resolution of more than one fibre orientation per voxel (Alexander et al., 2001). Given that the majority of voxels at typical ‘clinical’ image resolutions (e.g. voxels with dimensions of 2–3 mm), contain multiple fibre populations (Behrens et al., 2007; Jeurissen et al., 2012), this is a severe limitation.

Alternative methods such as diffusion spectrum imaging (DSI) (Wedeen et al., 2005), Q-ball imaging (Tuch, 2004), persistent angular structure MRI (PAS-MRI) (Jansons and Alexander, 2003) and spherical deconvolution (SD) (Tournier et al., 2004) have all been proposed to overcome the DT-MRI limitation. Data acquisition times for DSI are prohibitive, since sufficient data are required to reconstruct the full diffusion propagator. Q-ball imaging is a variant of DSI that, through the use of the Funk–Radon transform, allows peaks in the diffusion orientation distribution function (dODF) to be found from data acquired at a single b-value. However, peaks in the dODF are not very sharp in comparison to the fibre orientation distribution function (fODF), (Alexander and Seunarine, 2010); PAS-MRI and spherical deconvolution methods aim to obtain sharper estimates of the peak in fibre orientation, the former requiring extensive computation time due to non-linear estimation requirements while the latter class of techniques facilitate fairly rapid fODF retrieval, though peak finding may take considerably longer depending on the desired accuracy/speed trade-offs. As spherical deconvolution approaches: (1) attempt to recover the fODF directly (as opposed to the dODF); (2) have acquisition requirements similar to that required for robust DT-MRI (Jones, 2004); and (3) have reasonable computation costs, they have become the methods of choice in our laboratory.

The assumption underpinning most spherical deconvolution techniques is that an observed DW signal is the result of the spherical convolution of an underlying fODF with a response function that characterises the diffusion-weighted signal from a single-fibre population. Response functions may either be estimated on a voxel-by-voxel basis (Anderson, 2005) or, for simplification, assumed to be a constant across the image—i.e., a single response ‘calibration’ step is performed. The two most common calibration techniques are: (i) averaging measured signals in a region of high FA (e.g. FA > 0.8 Tournier et al., 2004); or (ii) by simulation of an idealised signal. Once a calibrated single fibre response function has been obtained, associated SD approaches proceed by deconvolving this response from the sampled DW signals (referred to here as the ‘target’) to estimate the underlying fODF. However, reconstruction of this fODF by spherical deconvolution is ill-posed and multiple solutions may exist, some of which may be physically implausible (such as negative peaks in the fODF). To address this, non-negativity (and typically non-small) constraints are often placed upon the deconvolution (Alexander, 2005; Dell'Acqua et al., 2007; Tournier et al., 2007) to improve fODF reliability.

Spherical harmonic deconvolution (SHD), and its constrained version (CSHD) are implementations of spherical deconvolution, proposed by Tournier et al. (2004, 2007), in which it is assumed that both the observed DW signal and the single fibre response functions may be adequately represented by a (truncated) linear combination of spherical harmonic basis functions. Typically the series is truncated at the 8th order harmonic, requiring a total of 45 coefficients to be estimated (Tournier et al., 2009). This both reduces the number of parameters that need be stored (reducing memory requirements) and, more importantly, reduces the deconvolution process to a computationally trivial operation in SH space (analogous to convolution/deconvolution in Fourier space), reducing overall processing requirements.

Where the assumption that all single fibre populations within an image volume exhibit the same diffusion profile is valid, CSHD can be shown to provide accurate estimates of fibre orientation (Tournier et al., 2008). In practice, however, there are many situations in which this fundamental assumption is not valid. For example, within healthy white matter, neuro-development (Suzuki et al., 2003) and normal inter-regional, inter-tract and intra-tract variations (Jones et al., 2005) can result in significant differences between diffusion profiles within an imaged volume. While a change in measured FA due to partial volume contamination will not necessarily alter the underlying diffusion profile, and a single profile assumption may still be applicable, regional variation of axon properties such as diameter/density (Abolitiz et al., 1992; Alexander et al., 2010; Barazany et al., 2009) or demyelination (Beaulieu, 2002; Gulani et al., 2001; Song et al., 2002) can result in genuine changes in the diffusion profile anisotropy (through increased radial diffusivity) of approximately 20%. This raises several important questions: Is it appropriate to use a single canonical fibre response function through the entire image volume? If not—what are the consequences of deviations from the assumed fibre response? While the assumption provides computational expediency, if there are deleterious consequences, should we consider sacrificing efficiency in favour of being robust against these deviations?

To address these questions, we first evaluate the performance of CSHD in the presence of a mismatch between the calibrated single fibre response and the fibre-response of the constituent fibre populations. We then compare the CSHD algorithm to dampened Richardson–Lucy deconvolution (dRL) (Dell'Acqua et al., 2010) which is one of a number of alternative SD techniques that has identical acquisition requirements to CSHD, but rather than using a spherical harmonic basis representation, dRL performs deconvolution across a super-sampled (interpolated) fODF. The potential advantage of the Richardson–Lucy algorithm is that, although a single fibre response calibration is still required, the Richardson–Lucy deconvolution framework is known to tolerate imprecision in the initial response function estimates (Dell'Acqua et al., 2007), potentially reducing the impact of imperfect response function calibrations.

We compare the performance of the two algorithms in the presence of a discrepancy between the calibrated and ‘target’ fibre responses (i.e., the DW-signal profile of the tissue in which the deconvolution process is to be applied). Specifically, we examine the impact of introducing a discrepancy between the anisotropy of the tissue used for calibration and the anisotropy of the fibres in the target tissue; examining signals representing both single and crossing fibre targets. While our motivation for the inclusion of crossing-fibre data should be self evident, our reasons for including the ‘simple’ single fibre configuration are two-fold: firstly, each time a new HARDI technique is developed, substantial efforts are expended to demonstrate its ability to recover the ODF in crossing-fibre configurations. Higher precision in the estimates of the orientation of constituent fibres and the ability to resolve smaller inter-population angles are the usual markers of efficacy. However, perhaps unsurprisingly, rarely do these investigations return to the seemingly trivial, yet essential, problem of reconstructing the ODF in single fibre populations. Such simple configurations are easily resolved by the techniques they aim to supersede (for example, the uni-modal Gaussian tensor model performs adequately in single fibre populations). Secondly, characterisation of any errors in the resolution of a single fibre orientation will provide an easier way of identifying any underlying systematic problems inherent in certain techniques, allowing us to diagnose their cause.

## Materials and methods

### Data simulations

Datasets were simulated (zero-mean Gaussian test function—Cook et al., 2006) assuming a typical 60 direction sampling scheme (Jones et al., 1999), b = 2000 s/mm^2^, and for four signal-to-noise ratios (SNR): infinite (i.e., noise-free), 50:1, 30:1 and 10:1 (consistent with Tournier et al., 2004). The sixty diffusion-weighted signals were computed for:

(i) a single, prolate, axially-symmetric tensor with fixed mean diffusivity (0.7 × 10^− 3^ mm^2^/s) and varying fractional anisotropy (FA) aligned along the x-axis;
(ii) a pair of ‘crossing fibres’ simulated as the signal produced by two prolate, axially-symmetric tensors the same FA, varying crossing angle (10–90°, 5° interval, one aligned along the x-axis, the other rotating about the z-axis) and signal contribution ratios (20/80% to 80/20%, 5% interval, first number represents signal contribution from the axially aligned fibre, second from rotated fibre)
(iii) a single, prolate, axially-symmetric tensor with fixed mean diffusivity (0.7 × 10^− 3^ mm^2^/s), fixed FA (0.3) and aligned along each of 752 axes evenly distributed on the unit sphere.

In simulation (i), when the SNR was infinite, the FA was varied between 0.1 and 0.9 at a 0.01 interval (81 individual profiles). For other SNR's in simulations (i) and (ii), the FA interval was increased to 0.1 and 500 repetitions of the noisy DW-signals were calculated per FA value (9 × 500 total). In simulation (iii), 500 noisy repetitions at SNR = 50:1 were generated for each of the 752 orientations (752 × 500 total). Using single-fibre ‘calibration’ responses from idealised (prolate axially symmetric) tensors with FA between 0.1 and 0.9, the peaks in the fODF and their associated magnitudes (fODFmag) were then extracted from all the simulated signals using both CSHD and dRL.

The noise-free data provides a ‘high resolution’ (81 × 81) array of fODFs, to gain a clear overview of the onset of any systematic failures that are not attributable to noise, while the noisy data allow assessment of the reproducibility of such artefacts at different SNR levels.

#### CSHD analysis

All CSHD analysis was performed using the algorithm exactly described by Tournier et al. (2007) and as implemented in the ExploreDTI software package (Leemans et al., 2009; see also Jeurissen et al., 2011). The harmonic series were truncated at the 4th, 6th and 8th orders (referred to as Lmax), requiring 15, 28 and 45 free parameters to fit, respectively, allowing us to explore impact of different truncations.

#### dRL analysis

All dRL analysis was performed using the algorithm described by Dell'Acqua et al. (2010) with deconvolution performed over 752 uniformly distributed fODF interpolation points (optional SH or radial basis function interpolation was not applied, and peaks were retrieved through an exhaustive search). A simple (cos^2^θ) response function with longitudinal and radial diffusivities of an axially symmetric prolate tensor with MD = 0.7 × 10^− 3^ mm^2^/s and the desired FA was used. Deconvolution was allowed to iterate 200, 300 and 400 times to explore the trade off between improving the angular resolution and over-fitting to noise within a runtime comparable to that of CSHD. Geometric and regularisation threshold parameters (Dell'Acqua et al., 2010) were set to 8 and 0.04 respectively.

#### DTI analysis

The principal eigenvector of the best-fit single tensor was derived from the raw DW signal data according to Basser et al. (1994).

### Data analysis—single-fibre, axially-oriented

The performance of the two SD algorithms was assessed according to three criteria: (1) the deviation between the peak of the reconstructed fODF and the principal eigenvector of the simulated tensor; (2) the magnitude and orientation of spurious peaks in the fODF; and (3) the expected ‘failure’ rate of implied fODFs if used by tractography algorithms.

#### Error in fibre orientation estimates

For a single fibre population, the orientation of the largest peak in the fODF (the ‘primary peak’) should provide the best estimate of the true fibre orientation. We therefore characterised the angular deviation between the orientation of the primary peak and the simulated fibre orientation for all calibration/target (C/T) pairings. We define the entire set of pairings as ‘C/T’ space. For the noise-free data, the comparison was straightforward. For the noisy data, for each SNR and C/T pairing, the average orientation of the 500 estimates of primary peak orientation was computed by taking the principal eigenvector of the associated scatter matrix along with the 95% confidence interval (Jones, 2002) to quantify orientational uncertainty.

#### Spurious peak manifestation and orientation

When using SD techniques to recover the fODF, the reconstruction of ‘spurious’ peaks is a common problem which is particularly problematic for tractography algorithms, i.e. to distinguish between artefactual and ‘real’ trajectories. To address this, the standard practice is to apply some form of threshold on the magnitude of the fODF peaks, so that only ‘large’ peaks are retained.

For the noise-free data (simulation (i)), our results were sufficiently dense (i.e., 81 × 81 pairings), that the maps of the magnitude of the spurious peaks were amenable to gradient edge detection, for which we employed the Canny operator (Canny, 1986) to identify and highlight boundaries in the C/T space.

To look for patterns in the relative orientation of spurious peaks with respect to the primary peak, for each C/T pairing, we first thresholded spurious peaks by magnitude (fODFmag > 0.1 for CSHD in keeping with standard literature practice, fODFmag > 10% of maximum for dRL). The spurious peaks were then visualised on the unit sphere and, as discussed below, were seen to fall into discrete clusters. K-means clustering (where k was selected on the basis of a visual inspection) was subsequently applied to extract mean angular orientations.

#### Expected tractography failure rate

To illustrate the practical implications of inappropriate calibration, we derived the frequency of occurrence of a ‘severe’ failure in a standard ‘Euler-like’ tractography algorithm (Basser et al., 2000). Here we define a severe failure as: (1) an incoming fibre trajectory subtending an angle with a supra-threshold spurious peak that is below any angular threshold used as a termination criterion (so that the tracking algorithm follows this spurious peak); (2) false negative or (3) false positive results where bias in fibre orientation causes erroneous fibre reconstruction.

To derive the frequency of these occurrences, we considered 100 uniformly distributed (Jones et al., 1999) axes to simulate tangents to possible incoming fibre trajectories. For each C/T/SNR combination (excluding noiseless data), the percentage of incoming trajectories resulting in a failure (as defined above) was defined for each of the 500 repetitions, and the mean computed. The angular threshold (i.e., the maximum angle through which the reconstructed streamline can turn between successive steps) was set to 35°.

### Data analysis—single-fibre, spherically distributed

The data from simulation (iii) allowed us to determine whether patterns in observed spurious fODF peaks (see the section ‘Spurious peak manifestation and orientation’) are orientationally variant. The target FA (T) was simulated at a fixed value of FA = 0.3, and calibration FA (C) was fixed to FA = 0.9—a combination known to produce spurious fODF peaks within the axially-aligned data. For each simulated fibre orientation we estimate the 95% cone of uncertainty in primary peak orientation (Jones, 2002), the mean number of spurious fODF peaks and the orientations of spurious peaks (fODFmag > 0.1) relative to the primary peak. These measurements provide insight into the orientational dependence of C/T discrepancy-driven uncertainty in the primary fibre orientation, and allow us to determine whether the manifestation of spurious peak patterns is a general phenomenon, or whether they appear more frequently for a given orientation (such as the orientation selected in simulation (i)).

### Data analysis—crossing-fibres

In the crossing-fibre case, performance was assessed according to two criteria: (1) the effect of miscalibration on angular resolution; and (2) the effect of miscalibration on sensitivity to differential fibre signal contribution (i.e., changing the relative compartmental volume fractions).

#### Angular resolution

A widely used performance metric for comparison of HARDI techniques is the minimum angle at which two distinct fibre populations may be reliably distinguished given comparable acquisition parameters, i.e., the angular resolution. To isolate the effects of C/T discrepancy we examined the subset of crossing-fibre data in which both fibres have equal FA and contribution to the diffusion-weighted signal, limiting the simulation variables to crossing angle, a single target anisotropy and calibration anisotropy. Here we regard a successful trial as resolving two supra-threshold fODF peaks (0.1 absolute magnitude CSHD, 10% of maxima dRL) within a 20° (Alexander, 2005) cone of error about the expected orientations.

#### Volume fraction

In the previous subsection it was assumed that both fibre populations contribute equally to the DW-signal. In reality this is unlikely to be the case and so we examined performance of the two algorithms as the compartmental volume fractions change. To simplify results, we examined an orthogonal crossing configuration with both fibres having the same anisotropy. The variable parameters were the relative volume fractions, the single target FA and the calibration FA. The same success criteria defined in the Section ‘Angular resolution’ was used.

### A word on fODF thresholding

Note that in some cases we applied an fODF threshold of 0.1 to CSHD results but none to dRL. From one perspective, this may be considered to be creating an unfair comparison (dRL may appear artificially worse). However, we adopted this practice for two reasons: (i) under miscalibration CSHD produces a large number of spurious peaks, however, a proportion of these peaks are of such insignificant magnitude that they will not survive the fODF thresholds typically applied in tractography algorithms, and would therefore be unlikely to cause any practical problems in tract reconstruction. Thus to present a fairer picture of CSHD for its real-world application in tractography, it is entirely appropriate to apply an fODF threshold. (ii) Conversely, almost all spurious peaks produced by dRL (against a single-fibre target) are insignificant in magnitude. By eliminating the threshold we are able to highlight any pattern of errors occurring with the dRL algorithm. For clarity, we clearly state where differential thresholds are applied.

## Results

### Single fibre orientation error: noise free data

**Fig. 1:**
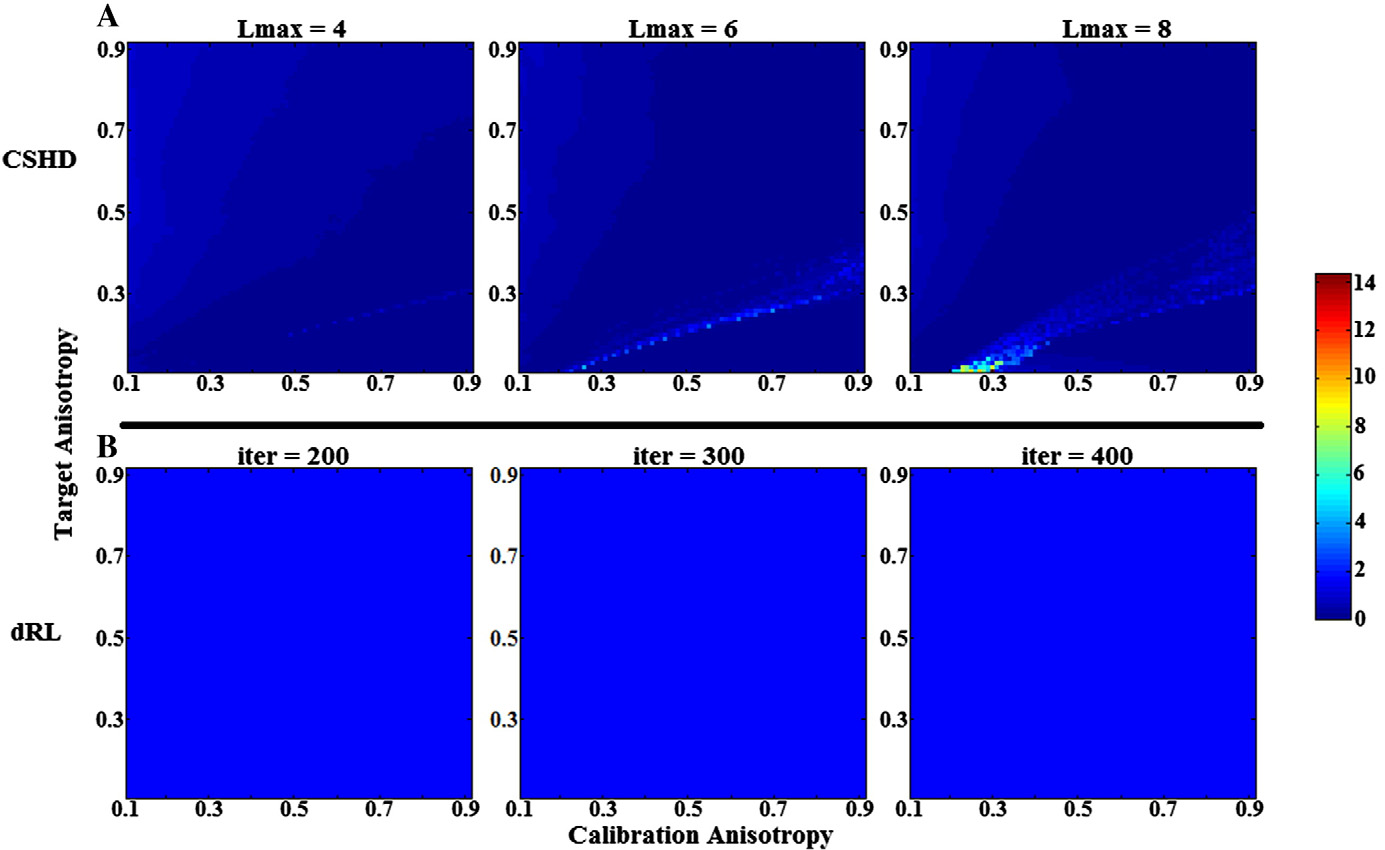
Angular bias (degrees) between the primary fODF peaks and simulated fibre orientations. A: CSDH. Note as Lmax increases a distinct region of increased angular bias begins to form (upper edge visible at C = 0.9/T = 0.5, lower edge C = 0.9/T = 0.3). B: dRL. Results demonstrate a consistent angular bias as a result of discrete sampling. Note that the horizontal axis depicts calibration anisotropy and the vertical axis depicts the target anisotropy, all subsequent figures will follow this convention.

Despite the absence of noise in the first set of simulated data (simulation (i)), both SD approaches produced erroneous results. The damped Richardson–Lucy algorithm results in a constant bias (angle between primary peak of the fODF and the simulated fibre orientation) of 1.13° (Fig. 1b) regardless of the number of iterations used in the deconvolution. This is consistent with the angle subtended between our simulated fibre orientation and the closest interpolated sampling point on the sphere, and is therefore an artefact attributable to the finite angular resolution afforded by discrete sampling (as compared to continuous differentiable functions, such as those used in CSHD).

Constrained spherical harmonic deconvolution, on the other hand, produces inconsistent errors, whose occurrence is dependent on the discrepancy between the calibration and target response functions (Fig. 1a). The majority of C/T pairings produce no error; it is only those pairings that lie between two distinct linear boundaries that lead to a significant bias in estimated fibre orientation (attenuated by reducing Lmax from 8 to 4). Note that instead of a horizontal boundary—which would imply that the bias is related to the target anisotropy alone, the bias in fibre orientation increases as the calibration discrepancy increases (i.e., calibration FA exceeding target FA).

### Single fibre orientation error: noisy data

**Fig. 2:**
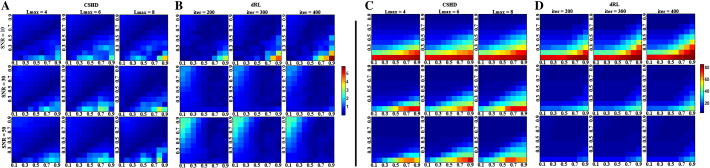
Left: bias in primary peak orientation of (A) CSHD and (B) dRL derived estimates of fibre orientation. Right: 95% confidence intervals in (C) CSHD and (D) dRL estimates or fibre orientation. Note that in all cases mean deviation for expected orientation remains low but uncertainty increases as C exceeds T.

Both SD algorithms produce noisy estimates of fibre orientation that are symmetrically distributed about the expected (simulated) orientation (Figs. 2a–b), suggesting minimal to no systematic bias in the estimate of primary peak orientation. However, CSHD (Fig. 2c) results do exhibit increasing uncertainty as the calibration disparity increases, and the uncertainty increases with an increased in Lmax. In contrast to the noise-free data, the number of C/T pairings was insufficient to facilitate a meaningful linear regression to identify boundaries in the results; however, visual inspection suggests that there is a similar linear boundary between affected and unaffected C/T space regions.

dRL results (Fig. 2d) show a similar trend towards increased uncertainty as calibration disparity increases, although the border between affected and unaffected regions does not appear linear and, perhaps more importantly, dRL appears to recover more quickly as SNR improves. It is interesting to note that, unlike CSHD, dRL results show an increased mean bias (though not uncertainty) in regions where T exceeds C and SNR remains high.

DT-MRI results (Figs. 4a–b) vary only with SNR and fibre anisotropy (reflecting the widely understood ‘noise bias’ (Jones, 2010; Pierpaoli and Basser, 1996a, 1996b). It is worth noting that at higher SNRs, in areas containing only single fibre populations with low anisotropy (0.1 ≤ FA ≤ 0.4), the uncertainty in estimates of principal fibre orientation is *lower* with DT-MRI than with either CSHD or dRL when the calibration discrepancy is large.

### Single fibre spurious peak magnitude

**Fig. 3:**
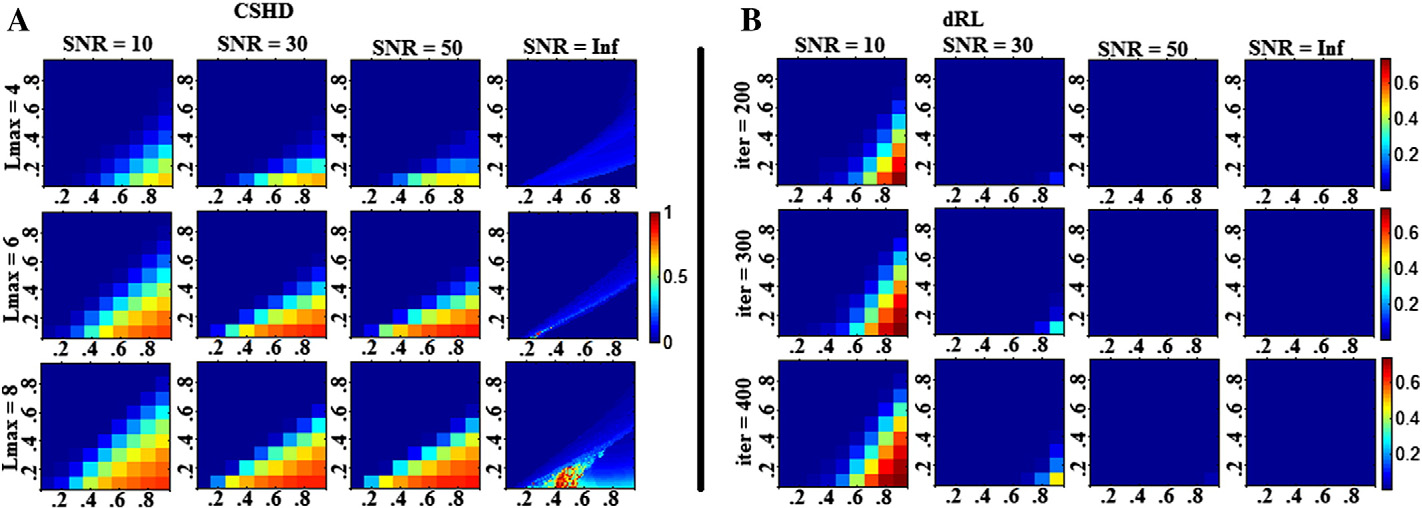
Mean magnitude of the largest spurious peak across data at SNR's of 10, 30, 50 and Inf. A: Results derived through CSHD. B: Results derived through dRL. While similarity exists between dRL and CSHD results at an SNR of 10, dRL (and Lmax = 4 CSHD) shows marked improvement as SNR increases which is not true for CSHD Lmax = 6–8. To facilitate an easier comparison, spurious peak values have been normalised by that of the related primary peak.

As might be expected, the appearance of spurious peaks in the CSHD-derived fODFs (Fig. 3a, SNR = ∞) coincides with the deviations in primary peak orientation (Fig. 1a). Regression of linear boundaries (at Lmax = 8) suggests that the magnitude of the secondary peak falls into three domains: (1) C/T pairings where T > 0.667C—spurious peaks exist but none are of sufficient magnitude to disrupt tractography algorithms; (2) C/T pairings where 0.667C > T > 0.5C—spurious peaks are discernible but mean magnitudes remain sub-threshold; and (3) C/T pairings where T < 0.5C—a substantial number of spurious peaks is produced (Fig. 8a) with sufficient magnitude to significantly corrupt results from tractography. The addition of noise (Fig. 3a) demonstrates that, as SNR decreases, the onset (with respect to calibration discrepancy) and magnitude of secondary peaks increases while following a similar pattern (linear boundaries between affected and unaffected regions). Lowering Lmax from 8 to 4 reduces the number of affected C/T pairings and the resulting spurious fODF peak magnitude (in both absolute and normalised terms—note Fig. 3 represents fODF data normalised by the primary peak magnitude).

Under noise-free conditions (Fig. 3b, SNR = ∞) dRL produces no spurious peaks. At high SNR, dRL will only produce spurious peaks under extreme C/T discrepancies, but, as SNR decreases, spurious peaks begin to form in regions where C > T. Increasing the number of algorithm iterations increases the number of C/T pairings which produce spurious fODF peaks and increases existing spurious peak magnitudes.

### Single fibre spurious peak orientations

Fig. 5a shows the elevation angles of CSHD-derived spurious peaks (thresholded fODFmag > 0.1, SNR = 50) plotted relative to primary peak orientation. These aggregated peaks (harvested from all C/T pairings) are not randomly distributed over the sphere, but instead demonstrate ‘banding’ with the number and location of each band dependant on both Lmax and, to a lesser degree, SNR (Table 1)—observations which also hold true for individual C/T pairings (Fig. 6a). It is important to note, however, that azimuthal values (Fig. 6b) do appear to be random in distribution.

In comparison to CSHD, dRL does not produce consistent banding patterns (Fig. 5b). While there is a slight preponderance for spurious peaks to occur at a 90° elevation at the lowest SNR (SNR = 10), the number of spurious peaks is greatly reduced as SNR improves, suggesting that these are noise-induced (rather than algorithm-induced). The reduction in number of spurious peaks with increasing SNR becomes more marked as the number of iterations in dRL is increased. It is important to remember that the dRL results were not filtered through an fODFmag threshold, which would eliminate the majority of the observed spurious peaks.

### Single fibre expected tractography failure rate

As might be expected, reducing the SNR increases the frequency of occurrence of severe failures in tractography (Fig. 7). Interestingly, however, while CSHD results show that an increase in SNR leads to a decrease in the number of C/T pairings likely to cause significant error (in line with previous results), the frequency of occurrence within affected regions of C/T space increases substantially as SNR increases, which appears counter-intuitive. However, while a lower SNR produces spurious peaks which are larger in magnitude (Fig. 3a), the absolute number of peaks per C/T pairing, on average, is reduced (Fig. 8a).

Assuming a roughly uniform distribution of spurious peaks (with respect to azimuthal angle, Fig. 6b), the ‘spherical surface area’ covered by a set of spurious peaks (and their associated 35° angular threshold ‘cone’) will be directly related to the size of the set. Thus, as the number of spurious peaks increases, so will the error rate. As Lmax is reduced, both the number of spurious peaks (Fig. 8a) and the number of elevational ‘bands’ in which they reside also decreases (to a single sharp band at 90° at Lmax = 4), reducing the potential surface area coverage and therefore explaining the reduction in error rates compared to those observed at Lmax = 8.

A different pattern is seen with dRL which, as previously shown, recovers to near noise-free results as the SNR improves, i.e. spurious peaks occur infrequently, with low magnitude (Figs. 3b, 5b and 8b), and with low uncertainty in the primary peak orientation (Fig. 2b). Even without thresholding, the expected error rates are substantially lower for dRL compared to CSHD (for comparable C/T pairings) in all but the lowest SNR case. Once again, however, increasing the number of algorithm iterations in dRL leads to a poorer result at high (C = 0.9) anisotropy calibration.

**Fig. 4:**
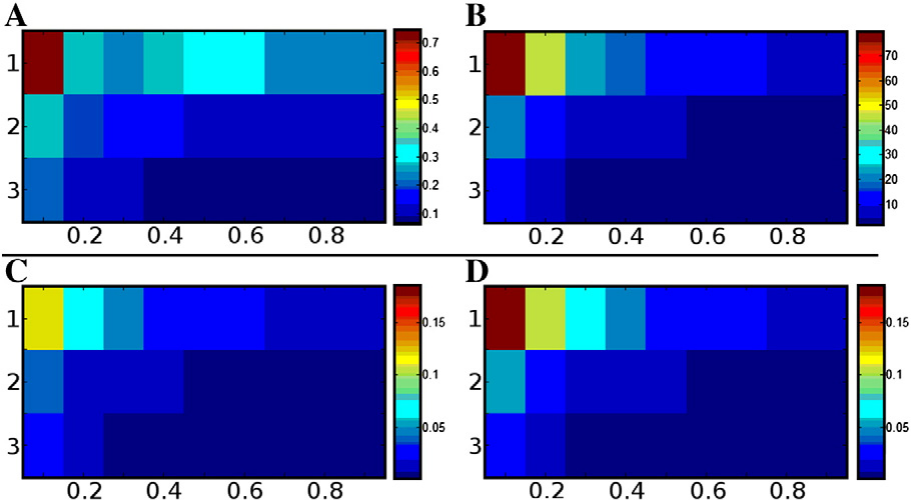
DTI performance. A: Error (degrees) in mean principal eigenvector orientation with respect to expectation (3 rows, row 1 SNR 10, row 2 SNR 30, row 3 SNR 50). B: Confidence intervals (degrees) about the mean principal eigenvector (A), note that at lower SNRs, only a small miscalibration is needed for DTI to begin to outperform CSHD (Fig. 2b) when resolving a single orientation. C: Expected tractography error (percentage [0 1]) at a 25° angular threshold while using DTI. While inferior to dRL at all but the lowest SNR/high miscalibration, only a small miscalibration is needed for DTI to outperform CSHD. D: Repeat of C at a 35° threshold, with similar results.

Given that the production of multiple spurious peaks through DT-MRI is an impossibility, (since there is only one principal eigenvector), failure rates (Figs. 4c and d) depend solely on the error in primary peak orientation. In this single fibre population case, DT-MRI results compare favourably with the HARDI alternatives, outperforming CSHD at low SNR/anisotropy once calibration discrepancy is introduced.

### Single fibre orientational variability

Fig. 9a indicates that for the majority of simulated fibre orientations, primary peak confidence intervals remain consistent with values predicted by similar axially aligned data (Fig. 3a). Note, however, that there is a marked increase in uncertainty for fibres aligned to the z-axis. This pattern is echoed closely by the mean spurious peak counts (Fig. 9b), the observed numbers closely match the axially-aligned data (Fig. 8) and variation (though small) appears to follow primary peak uncertainty.

**Fig. 5:**
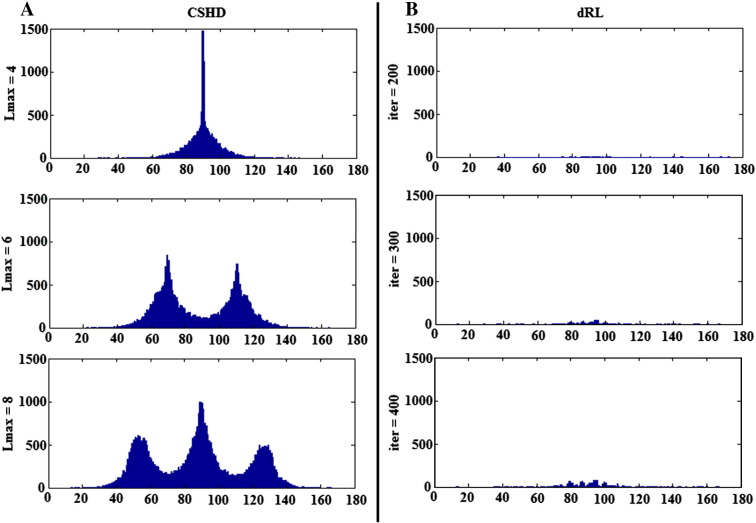
Aggregated elevation angles of artefactual peaks in SNR = 50 data. Left: CSHD. Right: dRL. Note the distinct structure within spurious CSHD peaks that shifts in relation to Lmax. For this figure CSHD peaks have been thresholded (fODFmag > 0.1), dRL results have not.

**Fig. 6:**
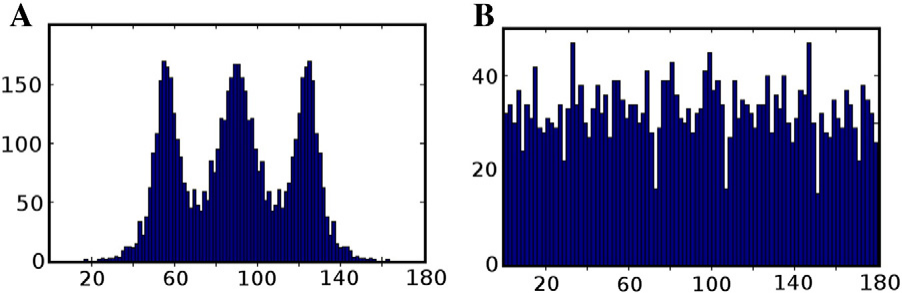
A: Distribution of spurious peak elevations (fODFmag > 0.1) relative to primary peak orientation (C/T = 0.7/0.3, SNR = 50, Lmax = 8). Note the concentrations about 54, 90 and 127°. B: Azimuthal values across the same data, notice that the distribution remains approximately even across the entire range (180–360° symmetry omitted).

We now focus on the relative distribution of spurious peaks (Figs. 9c–e). Fig. 9c contains the aggregate of all spurious peak orientations, and clearly shows the same characteristic Lmax dependent banding as seen in the axially-aligned data described in the Section ‘Single fibre expected tractography failure rate’ (Fig. 5), suggesting that the orientation of spurious peaks relative to the main peak orientation is independent from the simulated peak orientation itself. This can be confirmed by examining individual cases such as Fig. 9d—an example with typical primary peak confidence (fibres simulated along a [0.5774, − 0.5774, 0.5774] direction vector)—and Fig. 9e—a ‘high uncertainty’ example with z-axis alignment [0, − 0.1114, 0.9938]). In general the described banding patterns appear stable across all simulated fibre orientations, although there may be some orientational dependence of intra-band dispersion (i.e., bands appearing wider at certain orientations), the reason for this is unclear and remains a question for future study.

### Crossing fibre angular resolution

Two inferences can be drawn from the CSHD data (Figs. 10a and 11a–c): (1) optimal results are generally achieved by selecting a calibration that closely matches the target fibre (Fig. 10a). Underestimating target FA results in a gradual decline in angular resolution, while overestimation results in a sharp fall. There are exceptions, particularly at low SNR (Fig. 11a, SNR = 10, T = 0.9) where, for all but Lmax = 4, calibrations slightly lower than the target anisotropy yield better angular resolution; (2) increasing Lmax yields the expected increase in angular resolution, but does so at the cost of greater sensitivity to miscalibration. For example, at Lmax = 4 a calibration of 0.9 produces far fewer spurious results than at an Lmax of 6 or 8 (Fig. 11b). This is not because fibre orientations are unresolved, but—as in the single-fibre case—C > T pairings produce an abundance of spurious fODF peaks that foul the strict two-peak success criterion. Temporarily disabling this constraint (Fig. 13a) demonstrates this point more clearly as success rates for overestimated calibrations duly increase.

Figs. 11d–f highlight the differences between CSHD and dRL. As with the single-fibre configurations, a highly anisotropic calibration response function produces the optimal results in most situations. The one exception is at low SNR (Fig. 11d) where a (slightly) lower anisotropy provides more resilience to noise. Unlike CSHD, spurious peak formation only accounts for a low proportion of observed failures with dRL (generally this only happens at extreme calibration discrepancies at low SNR. Note that Fig. 13b shows improvement at SNR = 10) and is more likely to be due to failure to resolve more than one fODF peak or recovery of two peaks with large biases (Fig. 13c, but also notice minimal improvements in Fig. 13b—T = 0.6, SNR = 30). Finally, it is possible to improve the angular resolution of medium FA calibrations through increasing the number of algorithm iterations (Figs. 11d and e).

Overall, CSHD appears to be the superior choice for low FA targets (compare Figs. 11c with f), low SNR performance appears similar (Figs. 11a and d) and as SNRs and target anisotropies increase, the difference between best-case results is minimal. However, it must be noted that CSHD will require careful monitoring of target anisotropy to recover optimal results, while dRL generally will not. The figures referenced in this subsection are exemplars to demonstrate key results. A comprehensive set of results covering a wider range of C/T/SNR tuples is available in the supplementary material.

### Crossing fibre volume fractions

The results from varying the volume fraction are presented in Fig. 12. For CSHD (Figs. 12a–c), the best results are achieved through matching calibration to target anisotropy with the caveat that, at low SNR, a slightly reduced calibration anisotropy or lower Lmax may result in improvements (Fig. 12a, notice how C = 0.9 performance deteriorates as Lmax increases while the C = 0.7 calibration improves). Furthermore, increasing Lmax again allows one to resolve increasingly smaller signal contributions at the expense of requiring a more target specific calibration (compare C = 0.7/0.9 results at Lmax = 4 to Lmax = 8, Fig. 12b).

At low anisotropies, the performance of dRL again appears to be inferior to that of CSHD (Figs. 12d–f). This may be most clearly observed by comparing Figs. 11c and f, but subtle differences in performance are also discernible in Fig. 11R, where dRL performance is inferior in edge case performance (20/80, 30/70 volume fraction ratios) against correctly calibrated CSD (Fig. 11b). While low SNR performance remains comparable with CSHD, as with angular resolution, this is the one case in which a lower FA calibration may be preferable (Fig. 11D). As both SNR and target anisotropy increase it is possible to achieve best-case results through the use of a single C = 0.9 calibration. Though as with angular resolution, if necessary it is possible to achieve improvements using lower FA calibrations (Fig. 12d) or against lower FA targets by increasing the number of algorithm iterations (Fig. 12E, examine C = 0.9 as iteration count increases). Again, a more comprehensive result set is provided in the supplementary material.

## Discussion

**Fig. 7:**
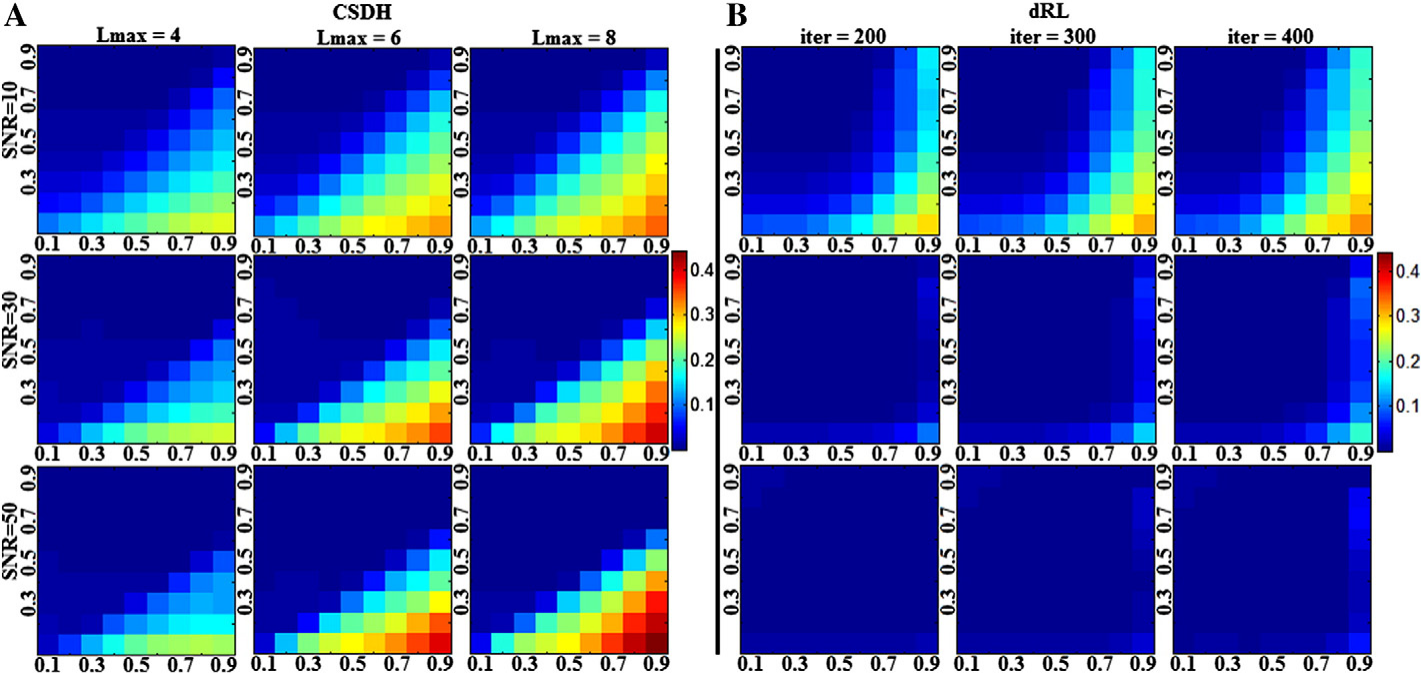
Approximate probability that an arbitrary incoming fibre trajectory will result in erroneous tractography through CSHD (A) and dRL (B) derived fODF estimates at a 35° angular tracking threshold. For this figure CSHD peaks were subject to a fODFmag > 0.1, dRL was unfiltered.

**Fig. 8:**
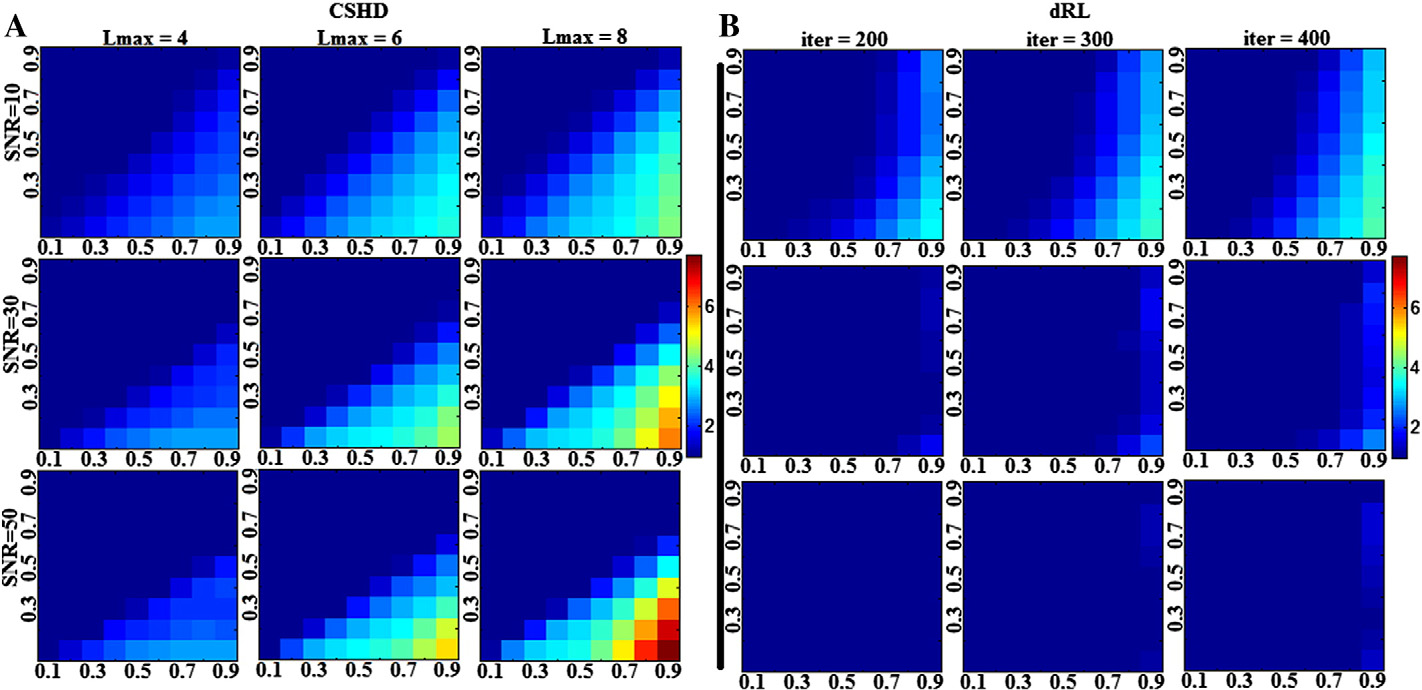
Mean number of supra-threshold (0.1 threshold applied to CSHD, 10% to dRL) spurious peaks observed through CSHD (A) and dRL (B). Note that with CSHD, as SNR increases, large (fODFmag > 0.1) spurious peaks occur later (requiring larger miscalibrations) but in greater numbers. The opposite is true of the dRL result, as SNR decreases the number of spurious peaks decreases towards optimal.

**Fig. 9:**
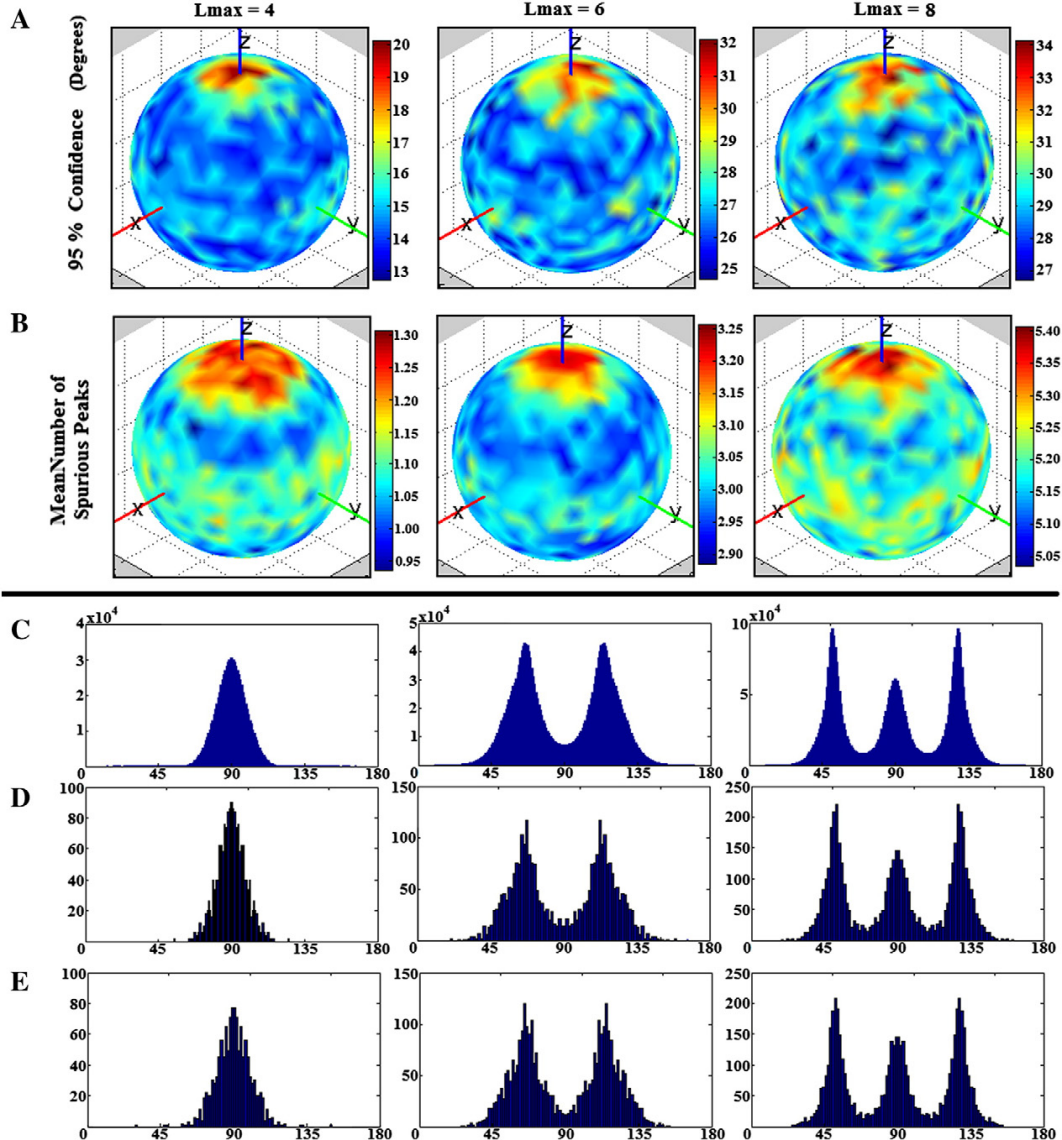
Impact of varying fibre orientation (simulation (iii)). (A) Plot of 95% confidence intervals for primary peak orientation (degrees). Note that there appears to be a pattern of increased uncertainty at along the Z axis which holds across the Lmax range. (B) A plot of the mean number of spurious peaks, note the correlation between A and B. (C) An aggregate distribution of all spurious fibre orientations relative to their primary peak. Notice that the distinct Lmax dependent banding appears to match axially-aligned results (D) Distribution of spurious peaks selected from a single fibre orientation chosen for its ‘typical’ 95% confidence interval and mean spurious peak count. (E) Distribution of spurious peaks selected from a single fibre orientation chosen for its a-typical 95% confidence interval and mean spurious peak count. Notice the distinct similarity between plots D and E.

Our results show that under the majority of circumstances, both CSHD and dRL will produce incorrect fODF estimates when calibrated to an inappropriate fibre response function (the exception being noise-free single-fibre dRL). It is clear that dRL, however, excepting the noisiest data or most extreme calibration discrepancies should not produce spurious peaks in sufficient quantity (Fig. 8) or magnitude (Fig. 3) to result in significant errors in tractography in regions containing single fibre populations (Fig. 7). However, in regions containing crossing fibres, dRL performance deteriorates when the target FA is low. CSHD, on the other hand, performs well across a range of target FA values in regions containing crossing fibres, but is prone to producing spurious fODF peaks in all cases as the calibration anisotropy begins to exceed that of the target.

Fig. 1, derived from noise-free CSHD data, shows a distinct linear boundary (T = 0.66C, Lmax = 8) between those C/T pairings that produce artefacts and those that do not. With the addition of noise (Fig. 2) it is seen that, on average, mean orientations are consistent with our expectation (simulated fibre orientation), and that individual estimates are distributed about the expected axis with no systematic bias. The result of calibration discrepancy therefore appears to be increased uncertainty in fibre orientation related to both SNR and the magnitude of the discrepancy. To illustrate the impact of varying the SNR in isolation, we can consider a toy example where the FA of calibration and target are identical (C = T), but the SNR varies. For C = T = 0.6 (Lmax = 8), the 95% confidence intervals in fibre orientation at SNR's of 10, 30 and 50 are 6.0°, 2.8° and 1.3° respectively. For C = 0.9, T = 0.6, placing the C/T pairing on the T = 0.66C border observed in noiseless data, the respective 95% confidence intervals are 14.4°, 8.5° and 5.7°, representing an approximate threefold increase over the noise-induced error. Thus the likelihood of experiencing calibration related artefacts at comparatively low calibration discrepancies is dependent on SNR. The presence of biases, however small, in the noise free data indicate that rather than C/T discrepancy simply amplifying noise-induced uncertainty, there are other systematic issues at hand.

Erroneous peaks lie in two distinct regions of interest in C/T space (Fig. 3c). Noiseless data predict that the first region is bounded by 0.667C > T > 0.5, here peak magnitudes/quantities should be lower (compared to the second region) and not seriously affect naïve tractography implementations. The second region defined by T < 0.5C should contain larger peaks in greater quantities and thus have more significant detrimental effects. The limited resolution afforded by the noisy data does not permit these same assertions; however, a general trend is seen where a reduction in SNR leads to an increase in the maximum magnitude and affected area (C/T combinations). Reconsider the T = 0.6 border case with ideal C = 0.6 calibration (Lmax = 8), it is seen that spurious peaks produced through noise have magnitudes of 0.055, 0.0291 and 0.0288 (for SNRs of 10, 30 and 50 respectively). Selecting a (C = 0.9; T = 0.6) miscalibration increases these magnitudes to 0.199, 0.126 and 0.088, placing two of the three peak amplitudes above the commonly-used 0.1 tracking threshold. We must therefore conclude that while underlying calibration related complications are responsible for the bulk of the artefacts (as evidenced by noiseless results and the persistence of artefacts at high SNR), calibration disparity may amplify pre-existing (yet harmless) artefactual peaks to disruptive magnitudes.

Finally, when examining expected tractography failure rates (Fig. 8), it must be remembered that our approach is naïve, in that only local effects are considered. For example, the ‘success’ criterion for an incoming streamline within an angular threshold of the expected orientation to continue is that the tangent to the streamline minimally subtends the best estimate of fibre orientation. What is not considered here is the impact that calibration-induced errors on the best estimate (up to 35°) will have on the following step; e.g. given even mild tract curvature, following such a trajectory may incorrectly cause a premature termination of the streamline by exceeding angular thresholds at the next step, or, initiate tracking down an erroneous path. Extending beyond the immediate locality, it is clear that calibration induced errors will have a more detrimental impact on tractography results than presented here.

### Orientation of artefactual peaks: a potential cause

To determine the cause of spurious peak formation, it is instructive to consider the basic mathematics underpinning CSHD. CSHD assumes that the fODF, single-fibre response function and DW-signal can be adequately approximated by a linear combination of spherical harmonics (Legendre polynomials). However, in order to reduce the computational load and the number of unique diffusion-weighted images that must be acquired, we tend to truncate the harmonic series to a predetermined maximum order—the property referred to as Lmax. While this is a prudent step to reduce data acquisition requirements and maximise model parsimony, there are some drawbacks. Although it is possible to sufficiently characterise the relatively smooth DW-signals and fibre response functions at low Lmax, characterisation of the sharper fODF often requires contributions from higher order harmonics whose omission leads to well known ringing effects (Anderson, 2005). In the single-fibre case, the ideal fODF response is a unit impulse with orientation along the fibre axis. However, attempting to describe this impulse with truncated harmonics results in a ringing artefact that generates both positive and negative spurious fODF lobes at predictable orientations relative to the impulse. By differentiating the responses at various Lmax, it is possible to find the orientation of the maxima of the positive ringing artefacts (note that negative lobes would be culled by the non-negative constraint implemented in CSHD). Table 1 and Fig. 5 show that orientations of the spurious fODF lobes observed in our empirical data are very similar to these theoretical values. We therefore consider that this simple artefact, exacerbated by miscalibration, is the root cause of the issues we are investigating. However, as the SNR decreases, there is an increased bias away from these predicted values, which is likely to be explained by the ‘squashed peanut’ phenomenon arising from the Rician noise floor, as described elsewhere (Jones and Basser, 2004).

Tournier et al. (2004) discuss the effects of deconvolving a wider diffusion profile from a narrower diffusion profile (in essence, C < T), stipulating that it may lead to production of negative terms in the diffusion fODF (although data were not provided). Essentially, we are exploring the opposite. By deconvolving a narrow diffusion profile from a wider one (C > T) we are erroneously adding, or increasing, positive terms in the resultant fODF thereby increasing the magnitude of the ringing induced fODF peaks beyond typical non-small/negative and fODF threshold constraints. It is not necessary to consider the original C < T case, since negative lobes are dealt with through the non-negativity constraint in the CSHD approach (Tournier, 2007), which explains the acceptable performance in these C < T C/T space regions.

It is also well-known that higher order harmonic representations are more sensitive to noise since the magnitudes of individual SH coefficients are reduced (Anderson, 2005); this may also explain variations with respect to Lmax. We notice that as we decrease Lmax, resilience to miscalibration improves. It may therefore be reasonable to consider miscalibration as analogous to noise; by performing a mismatched deconvolution we add or subtract from the coefficients of the ideal fODF result. Where ideal coefficients are large, miscalibration ‘noise’ will be small in comparison and thus only large miscalibrations will produce noticeable results (Lmax = 4). Where these coefficients are smaller, the ‘noisy’ contributions will have a larger relative impact, producing spurious fODF peaks at smaller miscalibrations (Lmax = 8).

Extending this argument allows us to begin to explain the counter-intuitive increase in spurious peak numbers with increasing SNR (Fig. 8). As calibration anisotropy varies with respect to the target diffusion profile, differences in diffusion profile width transition smoothly from negative to positive and, more importantly, distribution of such differences remains approximately equal at any given elevation—in effect creating a constant radial residual. The only way to describe this constant residual would be multiple ‘evenly’ distributed fODF peaks at elevations biased by the previously discussed ringing artefacts.

With the addition of noise, residuals at any given elevation are no longer constant, a diffusion weighted signal may indicate a higher than expected ADC along one orientation and a lower than expected ADC along another. The result (before the non-negativity constraint is applied) might be an fODF containing both negative and positive coefficients with much greater amplitudes than the comparable noiseless C/T pairing. In real terms this can mean that C/T pairs where T > C are able to produce positive/large fODF peaks (explaining the earlier appearance of spurious peaks at low SNR) and pairings where C > T are able to produce negative (explaining reduced numbers of spurious peaks) and larger positive (hence higher peak magnitudes at lower SNR) erroneous coefficients at the same time.

### A potential alternative: dRL

In practice, the assumption that the fibre response function can be adequately described through a truncated series is, while computationally elegant, potentially problematic. This is not to say that CSHD should be abandoned; in fact, crossing-fibre resolution performs well provided the calibration step is performed carefully. If, however, a narrow range of single-fibre responses cannot be guaranteed (breaking the assumption of a single canonical fibre response function), then alternative approaches may be of interest—of which dRL represents one possibility.

**Fig. 10:**
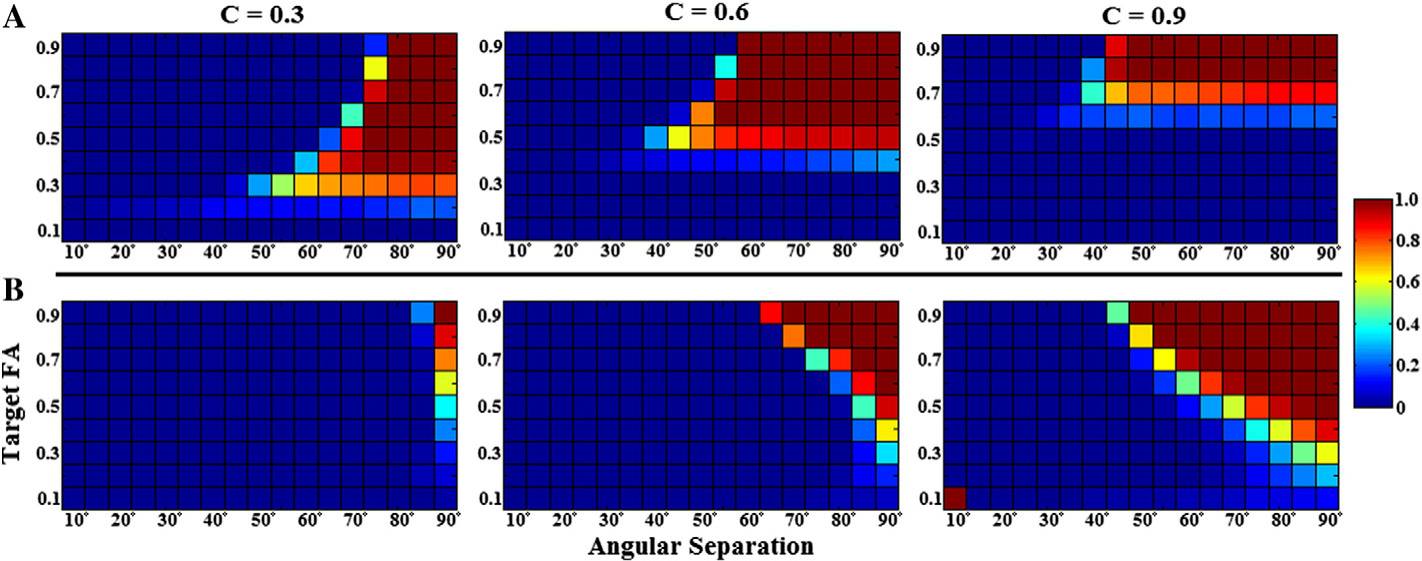
Crossing-fibre angular resolution: plot of crossing-angle (horizontal axis) against target FA (vertical axis) for SNR = 50 data over a range (C = 0.3, C = 0.6 and C = 0.9) of calibrations; colour indicates probability of successful fODF peak retrieval (scaled 0–1). (A) CSD results at Lmax = 8. Notice that best-case angular resolution is achieved at C = T then sharply drops as C exceeds T and gradually falls off as T exceeds C. (B) dRL results at iter = 200. Notice that for all cases C = 0.9 is the optimal choice, reduction in calibration anisotropy leads to a near linear reduction in angular resolution and there is a marked decrease in angular resolution as target anisotropy falls.

**Fig. 11:**
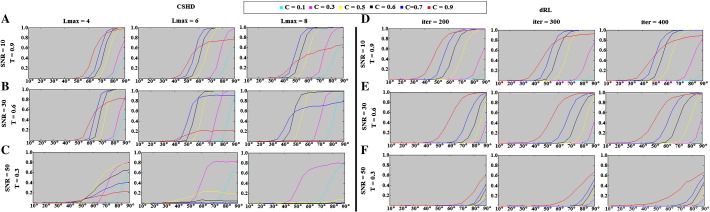
Crossing-fibre angular resolution: fraction of successful trials (vertical axis, parametrised between 0 and 1) vs. angular separation (horizontal-axis, degrees). (A) CSHD angular resolution at SNR = 10 vs. a T = 0.9 target. Note that as Lmax increases at this low SNR, the performance of the C = 0.9 (i.e. C = T) calibration deteriorates. (B) CSHD angular resolution at SNR = 30 vs. a T = 0.6 target. Note that best-case angular resolution increases with increased Lmax, the performance of C > T calibrations deteriorates indicating a trade-off between angular resolution and sensitivity to (over) calibration. (C) CSHD angular resolution at SNR = 50 vs. a T = 0.3 target. Note that when appropriately calibrated, CSHD is able to reliably resolve crossing fibre at low FA, compare this to dRL results (subsection F). (D) dRL angular resolution at SNR = 10 vs. a T = 0.9 target. Results are comparable to CSHD equivalents (subsection A); note that as the number of iterations is increased the angular resolution of C < 0.9 calibrations improves slightly at the cost of deteriorating C = 0.9 performance. (E) dRL angular resolution at SNR = 30 vs. a T = 0.6 target (matching subsection B). Note that unlike CSHD, dRL does not require a matched C = T calibration, instead C = 0.9 is (almost) always the superior choice. (F) dRL angular resolution at SNR = 50 vs. a T = 0.3 target. Note that C = 0.9 remains the best choice but performance is greatly reduced compared to appropriately calibrated CSHD (subsection B). A general pattern is observable across subsections D–F: A C = 0.9 calibration is optimal for most situations, though angular resolution can be expected to deteriorate steadily as a function of T.

**Fig. 12:**
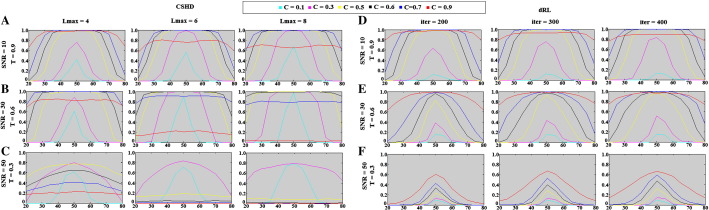
Crossing-fibre volume fraction resolution results: percentage of successful trials (vertical axis) vs. relative voxel contributions (horizontal axis, ticks denotes the percentage of the signal provided by the axially aligned fibre). (A) CSHD volume fractions resolution at SNR = 10 vs. a T = 0.9 target. Note that as Lmax increases at this low SNR, the performance of the C = 0.9 (i.e. C = T) calibration deteriorates while C = 0.6 and C = 0.7 calibrations slightly improve. (B) CSD volume fraction resolution at SNR = 30 vs. a T = 0.6 target. Note that while increased Lmax can improve best-case volume-fraction resolution (follow the C = 0.6 calibration), it does so at the cost of an increased sensitivity to miscalibration (follow C = 0.7 and C = 0.9). (C) CSD volume fraction resolution at SNR = 50 vs. a T = 0.3 target. CSHD seems more able to resolve volume fractions at low anisotropy than dRL in comparable situations (subsection F). (D) dRL volume fraction resolution at SNR = 10 vs. a T = 0.9 target. Best-case volume fraction resolution appears roughly consistent with CSD results. (E) dRL volume fraction resolution at SNR = 30 vs. a T = 0.6 target. Note that dRL tends to lose out at the edge cases (20/80, 30/70 splits) compared to CSD, though this is improved by increasing the iteration count. (F) dRL volume fraction resolution at SNR = 50 vs. a T = 0.3 target.

Single-fibre resolution through dRL is largely insensitive to calibration anisotropy and its performance should rarely be significantly worse than DT-MRI under similar noise conditions. Problems do however exist in the resolution of crossing-fibres. Our results show that in general dRL performs best with a high FA calibration, C = 0.9 providing the best results across the target anisotropy gamut. However, unlike the single fibre case, dRL's inbuilt damping is not always sufficient to suppress the formation of spurious peaks, leading to poor performance against low anisotropy, low SNR crossing-fibre targets at higher iteration counts. Unfortunately, unlike CSHD, one cannot simply select a matched (C = T) calibration. Much as a C < T calibration with CSHD will result in the loss of angular resolution, so will C < 0.9 with dRL (Figs. 10 and 11); the resultant fODF's remain overly rounded, leading to either a large imprecision in orientational estimates (biased towards the mean orientation) or a single peak (obviously this is not a problem for single-fibre targets).

**Fig. 13:**
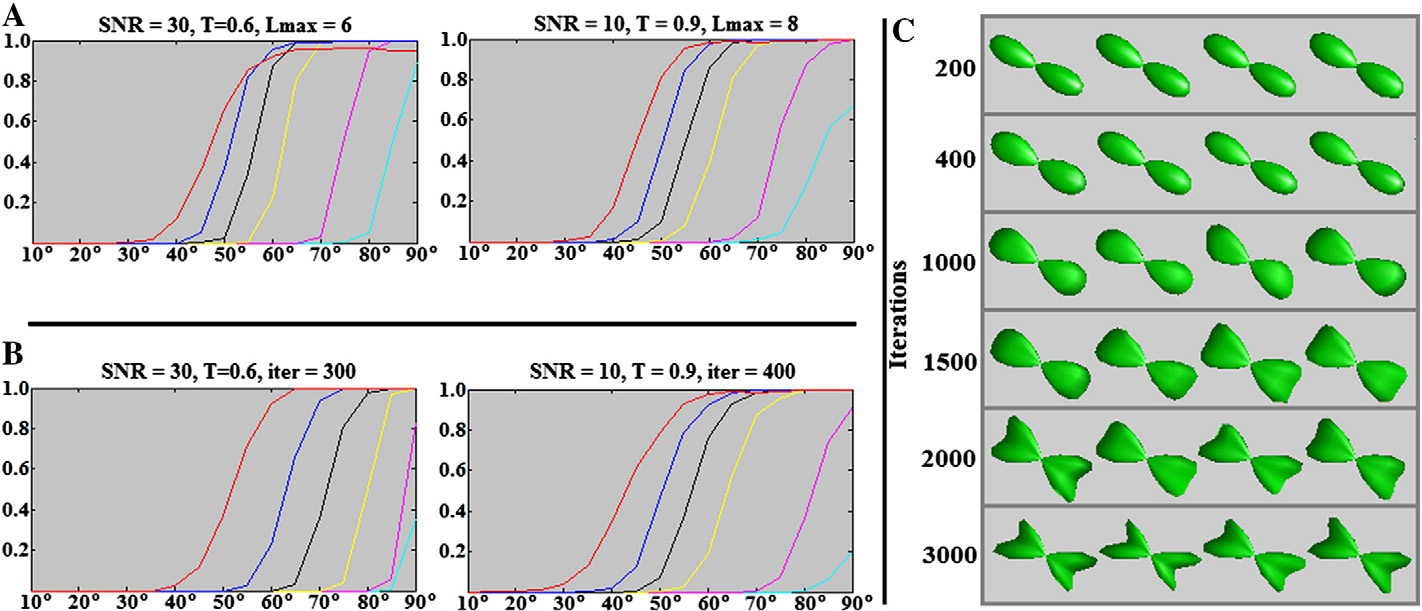
Angular resolution results if disregarding the “two peak only” success criteria—i.e. accept spurious fODF peaks provided the expected orientations are also recovered. (A) CSHD: notice that high FA calibrations (particularly C = 0.9) provide acceptable results in this circumstance. (B) dRL. Notice moderate/high SNR results do not change greatly from those with the strict criteria, however improvements are observed in cases with low SNR and high iteration counts. (C) dRL convergence for four examples of crossing-fibre, each consisting of two T = 0.9 target fibres crossing at 60° with 50/50 signal contribution and SNR of 50. Notice that with the selected C = 0.3 calibration dRL requires between 1500 and 2000 iterations to provide distinct indications of crossing fibre and nearly 3000 to resolve a ‘sharp’ fODF.

The main issue for dRL seems to be one of convergence. The algorithm is believed to return the best results shortly before the fODF deconvolution fully converges (Dell'Acqua et al., 2010), while actually reaching convergence can often result in an over-representation signals noise component; which can be most readily seen in single-fibre data (Fig. 7, C = 0.8/0.9, or the low SNR improvements Figs. 11d and 13b) where spurious peak formation causes error rates to increase with iteration count. The number of iterations required to reach the convergence ‘sweet spot’ varies as a function of both target and calibration anisotropies; as either decreases the number of iterations required to achieve an optimal solution increase—rapidly approaching computationally impractical levels (Fig. 13c) and becoming increasingly sensitive to target SNR in the process. This also explains the steady performance drop-offs with decreasing target anisotropy for a fixed number of iterations. As a toy example, it appears near impossible to reliably resolve T = 0.3 crossing-fibre fibres at C = 0.3 with an SNR of 10–20 regardless of the iteration count, while at higher SNRs it simply takes a long time.

To attempt to use dRL in this way also seems rather self-defeating. Committing to a C < 0.9 calibration will require an increased number of algorithm iterations which (ignoring the extra processing time which would likely make tailored CSHD a more tempting choice) will also likely preclude the ability to resolve higher anisotropy target fibres, crossing or otherwise, due to differential convergence points—i.e. a higher FA target will require fewer iterations to reach an optimal solution, the remaining additional iterations will most likely lead to spurious fODF peak formation due to previously discussed over-convergence effects.

In some ways it may be helpful to imagine the calibration properties of dRL as being ‘inverse’ to those of CSHD. In CSHD, a high anisotropy calibration response function will lead to spurious fODF peaks forming due to ringing artefacts, thus calibration at lower anisotropy is a better default position (avoiding the worst-case outcome and providing a graceful performance degradation as target FA increases). In dRL, however, a calibration response function with low anisotropy causes the spurious peak formation and a highly anisotropic calibration response function ensures the maximum number of iterations used will not exceed the over-convergence threshold for any possible target fibre, providing a graceful degradation as target anisotropy drops. Conveniently however, this default ‘high’ (C = 0.9) anisotropy is typically above anisotropies expected of ‘typical’ white matter (T > ~ 0.5) and thus, with practical algorithmic parameters, dRL can provide useful resolution of fibre orientations throughout the entire brain without the need for a carefully tailored calibration.

## Conclusion

The resolution of a *single fibre* orientation through SD methods is not as trivial as one may initially believe. While the addition of noise to a DW signal will obviously produce uncertainty in the estimation of fibre orientation and, if sufficiently severe, the appearance of spurious fODF peaks, we have shown a further confounding effect in the form of errors produced through inappropriate calibration. While dRL is exceptionally resilient to calibration error (in the single-fibre case), CSHD is particularly vulnerable to overestimation of the target diffusion profile regardless of DW-signal SNR. The truncated harmonic series response representation harbours descriptive deficiencies (ringing effects) which, as the magnitude of the calibration error increases, results in both predictable and unavoidable spurious fODF peaks.

That said, resolution of crossing fibres is equally important, but selection of an appropriate algorithm, calibration and running parameters is not a simple matter; trade-offs must be made depending on knowledge of the target image. For a white-matter target (FA > ~ 0.5) with no other *a priori* information, CSHD and dRL can be considered approximately equal. With CSHD it should be possible to achieve reasonable results with a moderate FA calibration in the 0.7–0.8 range (dropping Lmax if necessary) offering the best balance between preventing spurious fODF peaks at the lower FA range and maintaining angular resolution in the upper range. For dRL calibration is relatively straightforward, with C = 0.9 generally being the optimal choice and, while angular resolution is lost as the target FA decreases, resolution of single-fibre orientations will remain robust across the FA window—there are also other fringe benefits to dRL such as resilience to isotropic partial volume effects. If the range of target profiles is known, then the choice of algorithm is likely to be based upon the location of the damaged/low FA tissue of interest. If this occurs within regions of crossing-fibres, CSHD appears to be the solution of choice since: through tailored low FA calibration, it is possible to retrieve the highest angular resolution within the region of interest with accurate resolution of non-crossing fibres at a cost of reduced angular resolution/sensitivity to volume fractions in higher FA regions that may not be of interest. If, on the other hand, low FA tissue is non-crossing, then dRL would be the preferred choice. Resolution of crossing-fibre in high FA regions will be unaffected and, with a single-fibre target, low FA angular resolution issues are not a concern. For images where the diffusion profile remains constant, choice is largely dominated by the shape of that profile. For highly anisotropic diffusion there is little difference between dRL and CSHD, however, as profile anisotropy falls (e.g. to 0.3 for muscle, Parker and Jones, 2011), dRLs inability to refine a sharp fODF makes CSHD the preferred method, especially considering that it is possible to reduce Lmax for increased robustness at low SNR with little penalty in angular resolution at these anisotropies.

The real-world implications of these findings will probably not be felt by those studying healthy white-matter. However in white matter degradation or studies of other fibrous tissues, we have shown that choice of algorithm and calibration can have marked effects on the end result. This should be of particular interest to those currently complying with Tournier et al.'s (2004) calibration recommendation for CSHD, i.e. scanning the image raster-fashion for high anisotropy voxels (e.g. FA > 0.8) upon which to base an average fit calibration, where our results clearly demonstrate that a more considered calibration might lead to vast improvements in results.

In summary, the aim of this work was to highlight the effects of initial calibration on the veracity of two SD-derived fODF estimates. When presenting or reviewing a new HARDI technique, authors often (rightly) stress crossing fibre resolution performance but fail to address the seemingly trivial single fibre case; here, we have shown the pitfalls of such a strategy. Not only can the single fibre problem provide insight into a technique's strengths and weaknesses, it is also representative of a significant proportion of human white matter. Comparing DT-MRI to CSHD in particular, only a small C/T discrepancy is necessary for the performance of CSHD to significantly degrade compared to a ‘benchmark’ DT-MRI comparison (Figs. 2, 4 and 7). While we are not arguing that DT-MRI is superior to CSHD, DT-MRI's simple model and lack of an inherent *a priori* anisotropy assumptions does give it an inherent advantage with all single fibre signals (regardless of anisotropy) that must be closed through appropriate calibration. In essence, the advantages of crossing-fibre resolution are a definite boon, but utterly useless if single fibre resolution is incorrect (consider the consequences for the resulting tractography!). In practice it would be best if we could do away with these *a priori* image-wide assumptions in HARDI; indeed some SD techniques do in fact achieve this through voxel-wise modelling (Anderson, 2005). However, for dRL and CSHD several key recommendations can be made: provided diffusion profile anisotropy remains high (throughout this manuscript we have used FA as a parametrisation), both dRL and CSHD should provide accurate measurements of fibre orientation; dRL simplifies calibration choice (C = 0.9 is always optimal) while CSHD can be used to target specific locations with a tailored calibration. At profile anisotropies below 0.5, CSHD becomes the only choice, but the selected calibration anisotropy should be equal to, or slightly lower than, the lowest expected profile anisotropy; while this may cost in terms of angular resolution, it will avoid the ringing related artefacts that we have explored and single fibre estimates will remain robust.

The following are the supplementary data related to this article.

**Supplementary Fig. 1:**
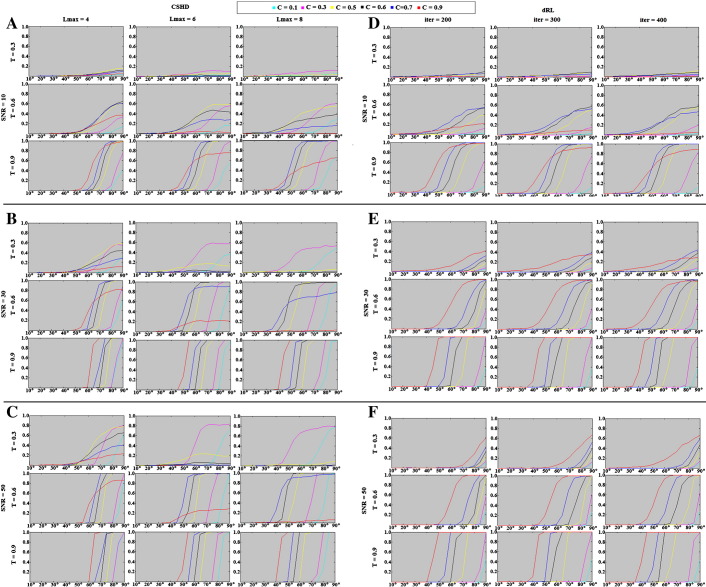
Crossing-fibre angular resolution: percentage of successful trials (y-axis, parametrised between 0 and 1) vs. angular separation (x-axis, degrees). A–C: CSHD results for varying Lmax at SNR's of (a) 10, (b) 30 and (c) 50. D–F: dRL results for varying numbers of algorithm iterations at SNR's of (d) 10, (e) 30 and (f) 50.

**Supplementary Fig. 2:**
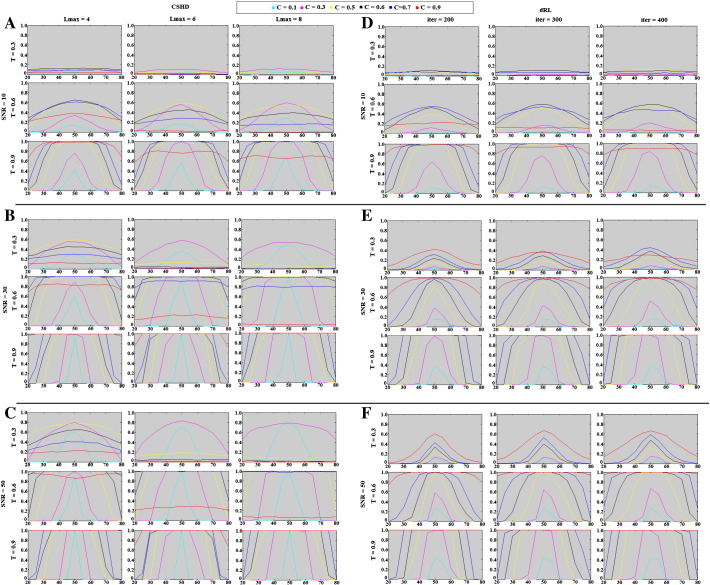
Crossing-fibre volume fraction resolution results: percentage of successful trials (y-axis) vs. relative voxel contributions (x-axis, ticks denotes the percentage of the signal provided by the axially aligned fibre). A–C: CSHD results for varying Lmax at SNR's of (a) 10, (b) 30 and (c) 50. 50. D–F: dRL results for varying numbers of algorithm iterations at SNR's of (d) 10, (e) 30 and (f) 50.

## Figures and Tables

**Table 1:** Mean elevation of cluster centroids in degrees (relative to primary orientation) of all artefactual peaks with fODFmag > 0.1 at SNR's of 10, 30, 50 with and Lmax of 4, 6 and 8. The general trend (as SNR improves) suggests convergence towards the elevations predicted by observing ringing artefacts in an fODF response approximating the unit impulse.

	Cluster 1	Cluster 2	Cluster 3
SNR 10			
Lmax 4	90°		
Lmax 6	71.4°	108.12°	
Lmax 8	59.49°	90°	120.06°
SNR 30			
Lmax 4	90°		
Lmax 6	69.8°	109.8°	
Lmax 8	56.77°	90°	122.46°
SNR 50			
Lmax 4	90°		
Lmax 6	68.75°	110.53°	
Lmax 8	54.44°	90°	124.87°
Prediction			
Lmax 4	90°		
Lmax 6	65.13°	114.89°	
Lmax 8	51.24°	90°	128.78°
